# Louis Pasteur

**Published:** 1904-09

**Authors:** Augustus A. Eshner

**Affiliations:** Philadelphia


### SELECTIONS AND ABSTRACTS.

### Louis Pasteur.

BY AUGUSTUS A. ESHNER, M.D., PHILADELPHIA.

In the list of benefactors of mankind, of those who have contributed in large measure to the welfare of their race, the name of Louis Pasteur will stand among the first. Of lowly ancestry, of humble, though sturdy, origin, this remarkable man, by training a physicist and chemist, succeeded by patient industry and persistent application in solving a series of problems in the field of biology, and thereby provided means that have resulted in the saving of untold lives and have contributed inestimably to the material prosperity of his native land and of all lands. He made important discoveries in crystallography, he helped destroy the doctrine of spontaneous generation, he took a large part in laying the foundation of the modern science of bacteriology, demonstrating the dependence of fermentation and putrefaction and of infection in human beings and lower animals upon the activity of microscopic forms of life, and he applied the principle of attenuation to the prevention and treatment of disease. All of these things he did, without hope or expectation of pecuniary reward, and with a devotion and unselfishness to which all other influences were subordinate. He was an example of the highest type of scientific investigator, systematic in method and critical to a fault, and in many ways he exhibited the prescience of genius. He maintained always an open mind, and, though not wanting in enthusiasm, permitted himself to utter no statement that he could not absolutely verify. He was, besides, an ardent patriot and a devoted son, husband and father. He was stricken with paralysis at the early age of forty-six, but undismayed by his disability, he resumed his work as soon as he was permitted by his physicians to do so, and for twenty-seven years more he directed a series of investigations resulting in incalculable benefit to the world at large. He died at the ripe of age seventy-three full of honors, and with an assured place in the temple of fame. The influence of his work will be felt throughout all time, and the labors he began are being carried on by devoted pupils in all parts of the world.

Pasteur was born in a small town of France, on December 27, 1822. His early ancestors were tillers of the soil, and serfs. His great-grandfather was a tanner, who purchased his freedom at the age of thirty. His grandfather and his father in turn also were tanners. The latter was carefully brought up, but not highly educated. He served in the French army under the great Napoleon, and finally became a sergeant-major, and was decorated with the Cross of the Legion of Honor. On leaving the army he resumed his trade. His wife was the member of an ancient plebian family. Louis Pasteur was the third child. He was a good average pupil and received several prizes in the lower grades of school. At thirteen he was said to be conscientious and studious, but without any apparent preference except for drawing. Already at this time he was noted, however, as a careful thinker and speaker, but with a vivid imagination. He was sent to Paris to school at sixteen but he became homesick, and was compelled to return, and continue his education at a local institution of learning. At eighteen he began to teach mathematics and physics, meanwhile continuing his studies. At the age of twenty he went again to Paris and entered upon the study of chemistry. At twenty-one he began investigation into the nature of tartaric acid, of which two varieties were known—ordinary tartartic acid, which turns the plane of polarized light to the right, and paratartaric or racemic acid, which turns the plane of polarized light to the left. Both exhibit the same crystalline form and the same atomic arrangement, but Pasteur showed that they are asymmetrical and hemihedral—that is, the facets of some of the crystals are directed toward the right while the facets of other crystals are directed towards the left. Separating the two varieties it was found that the one deflected the rays of polarized light in one direction, and the other the rays of polarized light in the opposite direction, while a mixture of the two in equal parts was optically inactive. Pasteur's studies in crystallography were continued for several years, and he did much to develop this department of knowledge. In the pursuit of his investigations into racemic acid he visited Germany and Austria. He eventually succeeded in producing for the first time racemic acid from tartaric acid, and also neutral or optical inactive tartaric acid or mesotartaric acid. The results of his studies along this line were embodied in a paper on dimorphism read in 1848.

At the age of twenty-four the prediction was ventured that Pasteur would make an excellent professor, and his forecast was in fullest measure realized. In the varied capacities of investigator, teacher, administrator, Pasteur ever rose to the occasion. At the age of twenty-six his laboratory studies were interrupted by his appointment as professor of physics at the Dijon Lycee. He devoted himself faithfully to the duties of teaching, although he longed to be back at his former work. Through the intervention of friends he was in the following year made assistant professor of chemistry in the University of Strasburg. Here he met his future wife, in the person of the daughter of the rector of the University, and whose hand he bespoke in marriage only two weeks after his arrival. His proposal was accepted, and he was married three months later. His domestic life though not free from sorrow, was in the main extremely happy, and in his wife he found a most willing and useful helpmeet.

In 1853 Pasteur was awarded the red ribbon of the Legion of Honor, and the same year he received a prize of 1,500 francs from the Pharmaceutical Society. Though possessed of but small means himself, he devoted one-half of the latter to the purchase of needed instruments for the Strasburg laboratory. In 1854 he was made a professor in the Faculte des Sciences at Lille, and dean. He was now located in a center of industrial activity, in a country of distilleries. Here he inaugurated a system of laboratory work on the part of his students, and with them he visited the manufacturing establishments of the neighborhood. For some time the local producers had encountered difficulty and suffered disappointment in the production of alcohol from beet-root, and for the purpose of discovering the cause and if possible providing a remedy Pasteur began his studies of fermentation. He showed that fermentation and putrefaction are dependent upon the activity of living organisms of low form, constantly present in the air, and that both of these processes can be prevented by exclusion of the exciting factors. His demonstrations gave the death-blow to the doctrine of spontaneous generation. His views, however, were not accepted without dissent, and numerous and vigorous were the discussions on both sides of the question. Even at this early date he began to appreciate the relation of atmospheric germs to disease. He observed that certain ferments thrive best when freely exposed to the air, while others, on the other hand, thrive best when cut off from the air, and he thus first made the distinction between aerobic and anaerobic micro-organisms. He recognized also the part played by germs in the destruction of dead organic matter with the aid of oxygen. In 1857 he was transferred to the Ecole Normale as administrative officer and director of scientific studies. Here, though not without difficulty, he organized a laboratory. In 1863 he entered upon the study of the diseases of wine, which had been the cause of much loss to his country. These also he demonstrated to be due to the activity of organized ferments, and preventable by exposure for a few moments to a temperature of from 50° to 60° C.—a process to which the name Pasteurization has been given.

In 1863 he was made professor of chemistry in the School of Fine Arts, and two years later he began his investigations into the diseases of silkworms, more particular pébrine, which had prevailed for twenty years, not alone in France, but in other countries as well, and threatened the very existence of the silk industry. He demonstrated that this disorder is dependent upon the activity of a parasite that attacks the egg, the worm, the chrysalis, or the moth, and by the conveyance of which the disease can be transmitted by contact. He succeeded in eradicating the blight by rejecting for purposes of propagation all ova found on the microscopic examination to be infected. In the course of his observations he discovered that another disease of silkworms—flacherie—likewise is due to the activity of a parasite that finds lodgment in the digestive cavity of the chrysalis. This disease also he eradicated by using for propagation only such moths as could be demonstrated microscopically to be free from the micro-organism.

In 1868 the honorary degree of Doctor of Medicine was conferred upon him by the University of Bonn in recognition of his studies in the field of bacteriology, but in the bitterness of feeling engendered by the Franco-Prussian war a few years later he returned the diploma.

In the year 1868, at the age of forty-six, Pasteur suffered a cerebral hemorrhage, with paralysis on the left side of the body, and it was for a time feared he would die. He improved, however, and in the course of three months he had resumed supervision and direction of further observations on the eggs of silkworms. To the genius of Pasteur must be awarded the credit of having saved the silk industry of France, as it had previously rescued the wine-industry. The intervention of the Franco-Prussian war interrupted his labors and sorely vexed his spirit. He deprecated the conflict of arms, but he would have gone to the front and done battle for his country had his infirmities not prevented him from so doing. He was an ardent patriot, and he developed a violent hatred for Prussia on account of what he considered a cruel and uncalled-for war of aggression. So intense was his feeling that, as already related, he returned the diploma that had been conferred upon him by the University of Bonn, and as late as 1895 he declined the proffer of a badge of the Order of Merit from the German Kaiser. By reason of the unsettled state of affairs in France at the close of the war Pasteur was offered laboratory facilities and a professorial chair in the University of Pisa, but he preferred to remain at home and aid in the rehabilitation of his country. He now entered upon a study of the causes underlying the spoiling of beer in the course of manufacture, and again he found these to reside in the activity of a fungus affecting deleteriously the yeast used at a certain stage in the process of brewing. Here also he showed that the undesired result could be prevented by Pasteurization.

Influenced by the discoveries of Pasteur showing that putrefaction and fermentation are due to the activity of germs, Mr. (now Lord) Lister began in the seventh decade of the last century that series of investigations that culminated in the development of his system of antiseptic surgery, and that in due course of time gave way to asceptic surgery. The principle underlying the method consists in destroying or rendering innocuous germs that may be present in and about wounds inflicted accidentally or made by the surgeon, and in preventing the further access of germs. These ends were attained by means of sprays and douches and protective dressings, in conjunction with the most rigorous mechanical and chemical cleanliness. The Germans and the French were slow to adopt the new practice, but it was not long before it became universal, and at the present time its neglect or omission is considerd, malpractice.

The belief that disease in man is entirely comparable to the fermentative and putrefactive processes grew constantly stronger in Pasteur. He demonstrated that ammoniacal fermentation of urine is due to a torula and could be prevented by addition of boric acid. He showed that wound-infections are due to bacteria, and out of his observations arose the practice of asepsis.

In 1874 Pasteur was awarded for life an annuity of 12,000 francs for distinguished and unselfish services to mankind, and this was increased to 25,000 francs in 1883 in recognition of the magnitude and enormous importance of his discoveries. In 1877 he took up the study of anthrax, the cause of which had already been shown by Davaine to be a special bacillus, and which was devastating the flocks of French shepherds and destroying considerable numbers of horses, cows, oxen, and even human beings. Pasteur, as well as Koch, succeeded in cultivating the bacilli on artificial media, and with the cultures reproduced the disease in lower animals by inoculation. He also developed anthrax in sheep by feeding them cultures of anthrax bacilli together with food capable of wounding the digestive tract. He found the spores of anthrax in soil contaminated by animals suffering from the disease, and he showed that the germs are brought to the surface by earthworms. In the course of these investigations it had been observed that anthrax fails to develop in inoculated animals previously treated with heated cultures of the anthrax bacillus. Pasteur demonstrated that this result is due to attenuation of the virulence of the parasites and not to their destruction. He found that such attenuation can be brought about also by the oxygen of the air and by the lapse of time, and that the virulence of the bacilli can be restored by passage through a series of lower animals. He was able to confer immunity upon animals by inoculation successively with cultures of gradually increasing virulence, and in this way he was the means of saving millions of francs to France. For his work in this connection he was offered the Grand Cross of the Legion of Honor, which he accepted only on condition that similar recognition be accorded his faithful collaborators, Chamberland and Roux.

In the year 1880 Pasteur cultivated on artificial media the germ of chicken-cholera, which had previously been discovered by others. He found that animals inoculated with old cultures survive if later inoculated with fresh cultures, and by this means he succeeded in lessening the ravages of this disease. In 1882 he rendered a like service in the case of swine-plague. At this time he took up also the study of hydrophobia. It was then, as now, believed that the virus of this disease is contained in the saliva. Inoculation experiments with this secretion, obtained from rabid animals, were undertaken, but the results were variable. As the symptoms of the disease are largely referable to the medulla oblongata, an emulsion of this part of the nervous system was made and employed for inoculation, and the results were now more constant. Still better results were obtained by inoculation beneath the cerebral dura mater. Pasteur was, however, unable to isolate the hypothetical micro-organism on which the disease is supposed to depend, and, in fact, no one else has as yet succeeded in that end. He was able, on the one hand, to intensify the activity of the virus by passing it through a series of rabbits, and on the other hand to diminish that activity by exposing the medulla of animals dead of rabies in a dry, sterile atmosphere for varying periods of time. It was found that the emulsion of a medulla thus exposed for a period of fourteen days could be injected without evil results, and consecutively emulsions of medulla exposed for a gradually diminishing number of days, down to the fresh medulla; and that finally animals thus treated were immune to the bites of rabid dogs and also to subdural inoculations.

It was now a question whether the results thus obtained in lower animals are applicable to man, and besides if the treatment is curative as well as prophylactic. Pasteur suggested to the Emperor of Brazil, who had addressed to him some inquiries on the subject that criminals condemned to death might be given the alternative of submitting to various experimental observations, and in the event of survival, which there was every reason to expect in the case of rabies for instance, have their punishment commuted to imprisonment for life, and he volunteered to go to Brazil to enter upon such an investigation under these conditions. As a result of his studies of rabies, Pasteur came to the conclusion that with the virus of the disease there may be associated a substance that through its influence on the nervous system renders this unsuitable for the growth and development of the supposititious microbe. The same principle he thought might be applicable also to other diseases.

The first antirabic inoculation in a human being was made in July, 1885, in the case of a boy nine years old, who had been bitten two days previously in many places by a rabid dog, and the result happily proved successful. From now on there was a steady list of applicants for the treatment, and all went on well until a girl of ten came thirty-seven days after having been severely bitten by a mountain dog. Pasteur hesitated to undertake the treatment in this case, but he could not refuse, and the child succumbed on the twenty-third day. Among those sent to the laboratory for treatment were four children from the United States, together with their physician, and whose expenses were defrayed out of a fund collected by the *New York Herald.* Happily all four recovered. Contributions began to pour in from numerous sources to carry on the good work. Thus far there had been only one death among the three hundred and fifty subjected to the treatment, as compared with a previous mortality of 16 per cent. upon a conservative basis. Of nineteen Russians badly bitten by a rabid wolf two weeks previously, all but three recovered. The Czar of Russia made a gift to Pasteur of a cross set in diamonds, and he donated 100,000 franks for the purposes of the proposed Pasteur Institute. A distinguished English commission investigated the method of treatment and the reported results, and after a study covering a period of fourteen months sustained all of the claims made. Pasteur's pupils now undertook the publication of the *Annals of the Pasteur Institute,* which it was intended should constitute a record of the work done in the laboratories.

On October 23, 1887, Pasteur suffered a temporary loss of speech, from which he recovered, only to suffer a repetition. In 1888 the Pasteur Institute, erected at a cost of one and a half million francs, out of a fund of two and a half million francs, raised by subscription, was formally dedicated to the purposes of the treatment of hydrophobia, of the study of infectious diseases, of the manufacture of vaccines, and of teaching. Pasteur Institutes have since been established in many parts of the world for similar purposes.

On December 27, 1892, Pasteur celebrated his seventieth birthday, in the presence of the President of the French Republic, the President of the Senate and of the Chamber, ministers, ambassadors, official delegates, and representatives from many countries. Some of his pupils have made important studies in connection with dipththeria. Two of them, Roux and Yersin, isolated the toxin of the disease, and the former produced the antitoxin almost simultaneous with Behring and Aronsohn in Germany. Yersin made a study of plague in China, and showed that the disease is conveyed by rats and flies. Both he and Kitasato discovered the plague bacillus at about the same time. Another pupil of Pasteur's, Metschnikoff, has made important contributions to our knowledge of phagocytosis and immunity. Another, Calmette, has made an extensive study of snake-poisoning, and has produced an antidote by the treatment of animals with venoms of various kinds. Still another, Haffkine, has successfully treated large numbers of persons in India with a serum protective against cholera.

Pasteur was seized with uremia in November, 1894. He recovered, but death overtook him September 28, 1895, at the age of seventy-three. The master is dead, but his work survives and will live permanently.

